# Systematic identification of a panel of strong promoter regions from *Listeria monocytogenes* for fine-tuning gene expression

**DOI:** 10.1186/s12934-021-01628-w

**Published:** 2021-07-12

**Authors:** Qianyu Ji, Junfei Ma, Shuying Wang, Qing Liu

**Affiliations:** School of Medical Instrument and Food Engineering, University of Shanghai for Science and Technology, Shanghai, 200093 People’s Republic of China

**Keywords:** *Listeria monocytogenes*, Promoters, RNA-seq, Exogenous protein

## Abstract

**Background:**

Attenuated *Listeria monocytogenes* (Lm) has been widely used as a vaccine vector in the prevention and treatment of pathogen infection and tumor diseases. In addition, previous studies have proved that the attenuated Lm can protect zebrafish from *Vibrio* infections, indicating that the attenuated Lm has a good application prospect in the field of aquatic vaccines. However, the limitation mainly lies in the lack of a set of well-characterized natural promoters for the expression of target antigens in attenuated Lm.

**Results:**

In our study, candidate strong promoters were identified through RNA-seq analysis, and characterized in Lm through enhanced green fluorescent protein (EGFP). Nine native promoters that showed stronger activities than that of the known strong promoter P_36_ under two tested temperatures (28 and 37 °C) were selected from the set, and P_29_ with the highest activity was 24-fold greater than P_36_. Furthermore, we demonstrated that P_29_ could initiate EGFP expression in ZF4 cells and zebrafish embryos.

**Conclusions:**

This well-characterized promoter library can be used to fine-tune the expression of different proteins in Lm. The availability of a well-characterized promoter toolbox of Lm is essential for the analysis of yield increase for biotechnology applications.

**Supplementary Information:**

The online version contains supplementary material available at 10.1186/s12934-021-01628-w.

## Background

 As a facultative anaerobic food-borne pathogen, *Listeria monocytogenes* (Lm) can cross the intestinal barrier, blood-brain barrier, and placental barrier of the host, leading to gastroenteritis, meningitis, and sepsis [1]. After Lm infects the host, it can use a series of virulence factors, such as those expressed by the *hly*, *actA*, and *plcB* genes to colonize and survive in the cell, thereby evading the humoral immune response of the host [2]. Lm can be used as an antigen-presenting carrier due to its unique viability in the cytoplasm of antigen-presenting cells [3]. At present, attenuated Lm has been widely used as a vaccine vector in the prevention and treatment of tumor diseases, such as liver cancer, cervical cancer, and pancreatic cancer [4–7]. It is worth mentioning that Advaxis, a clinical-stage biotechnology company, has made a major breakthrough in Lm immunotherapy technology. Among them, the ADXS11-001 vaccine, a live attenuated Lm bacterium bioengineered to secrete the HPV-16 E7 protein fused with a truncated fragment of listeriolysin O (tLLO), has been used in the treatment of anal and cervical cancer and has conducted clinical trials [8, 9]. In addition, the ADXS31-142 (secretion of LLO and human prostate specific antigen) and ADXS31-164 (secretion of chimeric tLLO fusion protein containing immunogenic regions of HER2 protein) vaccines were used to treat prostate and breast cancer, respectively [10, 11].

 The attenuated Lm has been successfully applied to mammalian models as a vaccine vector and has shown potential application in aquatic vaccines. Attenuated Lm with multiple variations is obtained by knocking out different virulence genes of Lm, with *inlB* and *actA* being the most frequently investigated. The *inlB* gene mediates the internalization of Lm to different cells such as hepatocytes, and the *actA* gene mediates the actin-based intracellular motility of Lm [12]. Therefore, knockout of these two genes can help reduce liver toxicity of Lm and block its spread. Our previous work revealed that attenuated Lm could induce an immune response in zebrafish. Activation of TLR signaling pathways may play a major role in the innate immune response [13]. In addition, we found that attenuated Lm could protect zebrafish from the infection of *Vibrio* species. We selected the outer membrane protein K (OmpK) from *Vibrio parahaemolyticus* as a protective antigen. After intraperitoneal administration in zebrafish, Lm EGD-e △*actA/inlB* and Lm EGD-e △*actA/inlB*-OmpK strains improved the survival rates of zebrafish infected by *Vibrio parahaemolyticus*, *Vibrio alginolyticus* and *Vibrio anguillarum*, respectively [14]. However, the expression level of heterologous antigens in Lm needs to be improved. Promoters play a key role in the expression of the target antigen as an important regulatory element. Promoters with high activities that can regulate the expression of genes in Lm are still very infrequent. So far, only the promoter P_help_ (named P_36_ in this study), has been described and shown to be a strong promoter in Lm. P_36_ was generated by introducing the 5’ untranslated region of the Lm EGD-e *hlyA* gene into a constitutive lactococcal promoter P_CP25_ [15]. To address the limitation, a panel of putative promoters with different transcriptional strengths has been systematically obtained via RNA-seq based transcriptional profiling [16]. For example, Kong et al. identified strong native promoters in *Streptococcus thermophiles* based on the above method, and these promoters have the potential to synthesize natural products [17].

In this work, 28 and 37 °C were selected as the growth temperatures to obtain RNA-seq data of Lm EGD-e. The most common growth temperature for Lm EGD-e in lab settings is 37 °C [14, 18–20]. A suitable growth temperature for fish, including carps and zebrafish, is 28 °C [21, 22]. The strong promoters selected under this temperature would have the potential to be used in the application of aquatic vaccine vectors. Based on the RNA-seq analysis of two tested temperatures, we identified 30 candidate promoters from Lm EGD-e. Subsequently, these 30 candidate promoters were cloned upstream of the reporter gene EGFP, and only 28 promoters were successfully cloned. Nine of them showed stronger activities than other promoters tested at 28 °C. Therefore, these promoters are expected to be used in the construction of attenuated Lm live vector vaccines so that they can be applied in the prevention and control of aquatic diseases. At the same time, the 9 promoters also showed strong activities at 37 °C, which can be applied to the study of attenuated Lm vaccines in mammalian animal models [23, 24]. The activity of the promoter P_29_ in ZF4 cells and zebrafish embryos was evaluated to further determine the feasibility of its application as aquatic vaccine vectors. The expression of EGFP in Lm could be observed under a fluorescence microscope through the invasion experiment of Lm EGD-e △*actA/inlB* (pERL3-29) in ZF4 cells. Moreover, the expression of EGFP in zebrafish embryos could also be observed after 26 h of microinjecting Lm EGD-e △*actA/inlB* (pERL3-29) into zebrafish fertilized eggs.

## Results and discussion

### Screening of strong potential promoters in Lm EGD-e via RNA-seq

**Fig. 1:**
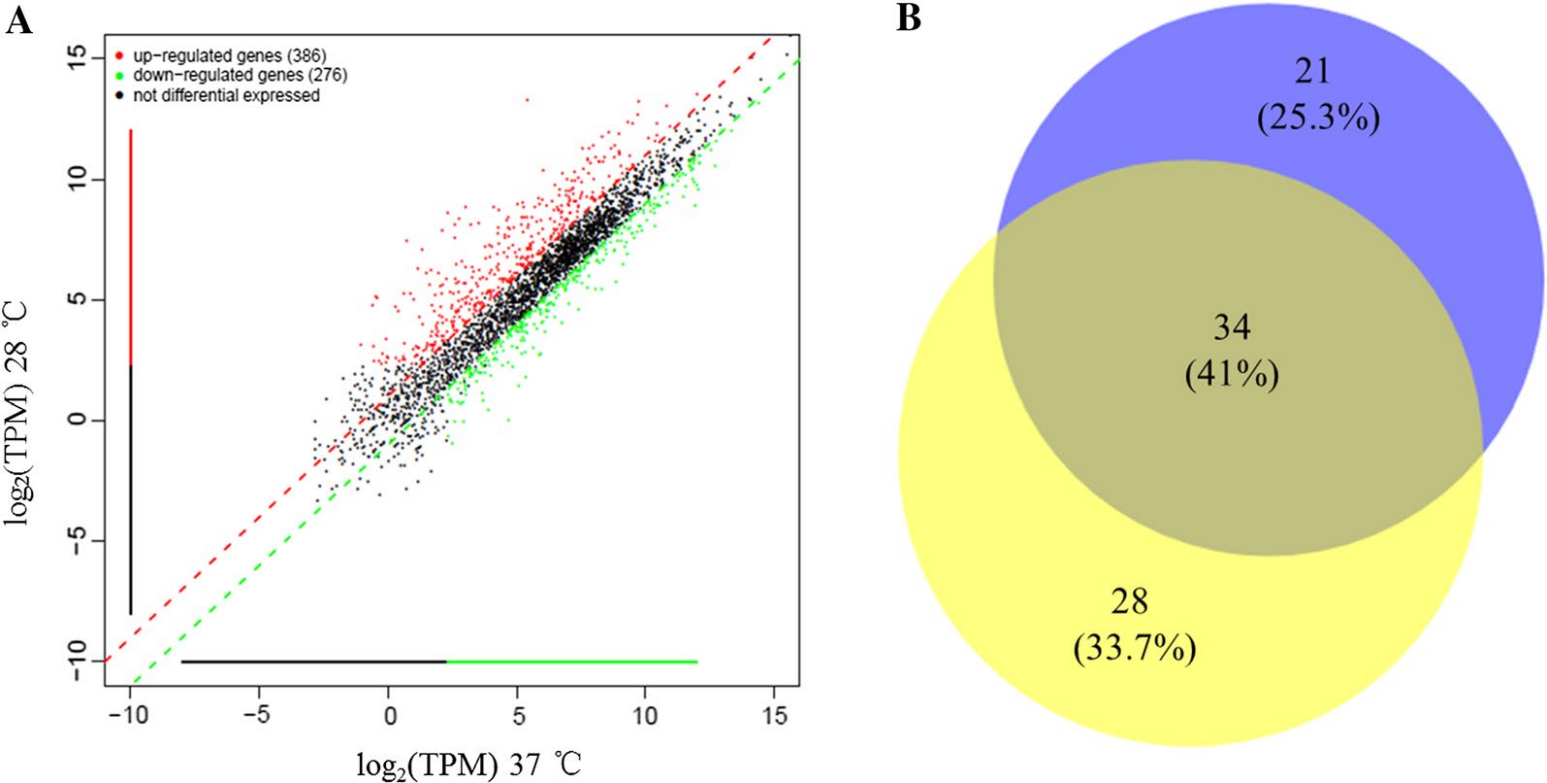
Screening of strong putative promoters from Lm EGD-e via RNA-sEq. **A** Scatter-plot of the expression level of genes or transcripts at the 28 and 37 °C by RNA-sEq. **B** Venn diagram of the number of highly expressed genes under 28 °C (blue) and 37 °C (yellow) by RNA-seq

Lm grown at 28 and 37 °C respectively were used to identify promoters driving high expression levels of native genes via RNA-sequencing data. The raw sequencing data was deposited in SRA database (NCBI Accession PRJNA718706). The transcription levels of all 2952 genes were analyzed for each temperature (28 and 37 °C, Fig. 1A). Transcripts Per Million (TPM) measures the proportion of a certain transcript in the RNA pool. The TPM values of genes higher than 3000 (near the top 2 % of highly expressed genes) were considered highly expressed. Genes of each sample were ranked according to the TPM value from highest to lowest. The TPM values of 55 genes and 62 genes were higher than 3000 under 28 and 37 °C respectively. The transcription levels of 34 genes were high under the two tested temperatures (Fig. 1B). Therefore, promoter regions were selected from these 34 highly expressed genes.

### Cloning of promoter regions identified through RNA-seq

**Fig. 2:**
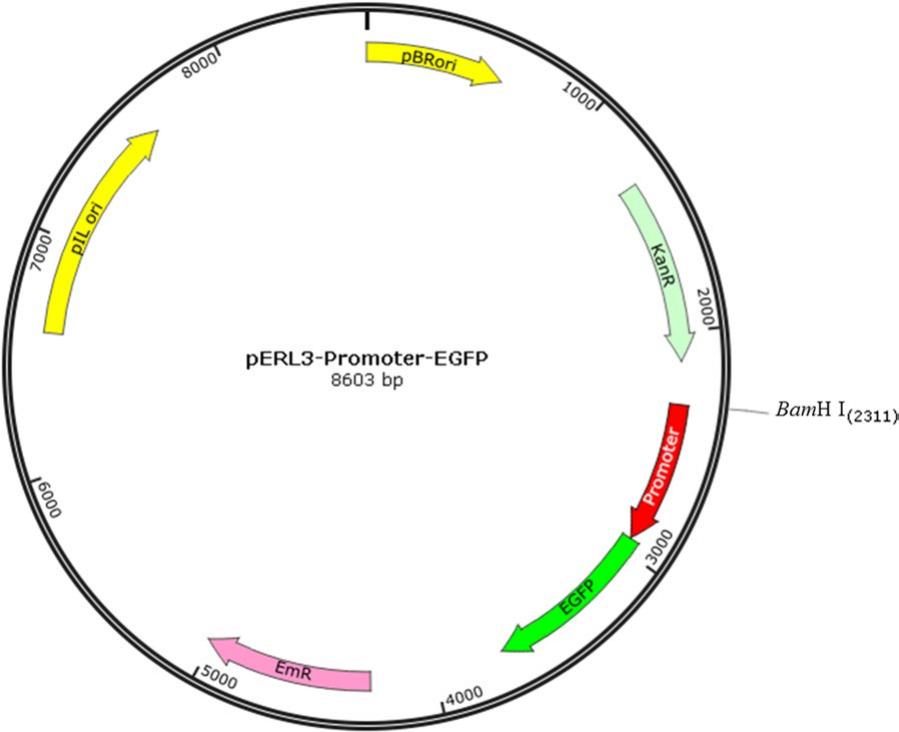
Schematic diagram of constructed plasmid pERL3-Promoter-EGFP. The coding sequences of promoter regions and EGFP were cloned into the *Bam*H I site. The promoters were used to express the EGFP reporter gene. pIL ori allowed for expression in Lm, and pBR ori allowed for expression in *Escherichia coli* [24]. The restriction site was *Bam*H I

Among these 34 genes, 4 genes were in the same operon. Therefore, only promoter regions of 30 candidate genes and 2 control genes were chosen for cloning. Intergenic sequences between the highly expressed gene and its upstream gene were selected to clone as the promoter regions. The Lm and *E. coli* shuttle vector pERL3 was digested with *Bam*H I, and the coding sequences of promoter regions and enhanced green fluorescent protein (EGFP) were cloned into the *Bam*H I site (Fig. 2) [24]. The resulting constructs were identified by PCR. Then, the sequences of the resulting constructs were sequenced to confirm the accuracy of the constructs. Twenty-eight promoters were successfully ligated with EGFP and transformed into Lm. See Additional file 1: Table S1 for details on plasmids.

Two constructs (pERL3-35 and pERL3-36) were built as positive controls. P_35_ is the promoter of *hly* in Lm. The *hly* gene encodes an important virulence factor of Lm named listeriolysin O (LLO), and it can make Lm escape the phagosomes [12]. P_35_ has been used previously in the construction of attenuated Lm expression vectors [14, 24]. P_36_ is a strong promoter in Lm constructed by Riedel et al. [15] who improved a luciferase tagging system for Lm by using P_36_. The results showed that P_36_ gave 100-fold-higher activity in broth than other native promoters tested [15].

### Characterization of the chosen promoters using the EGFP reporter gene

**Fig. 3:**
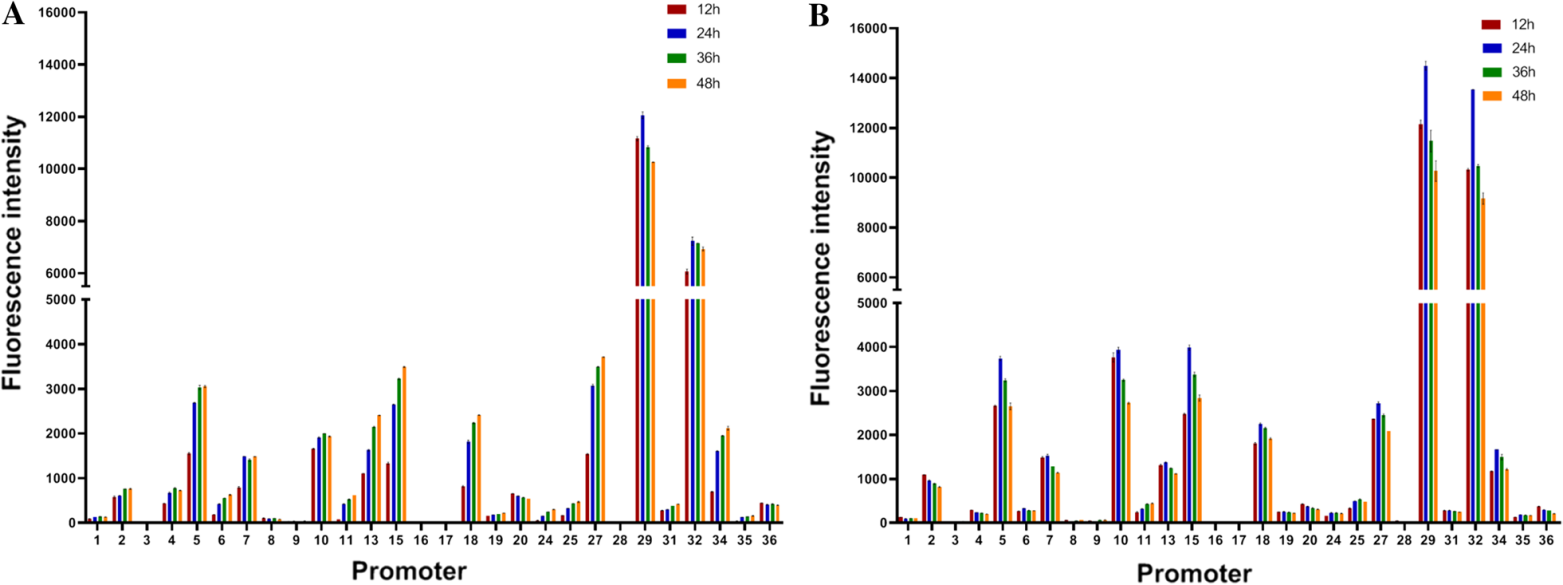
The strength of the 28 promoters. **A** EGFP expression by different putative promoters in Lm EGD-e under 28 °C. **B** EGFP expression by different putative promoters in Lm EGD-e under 37 °C. Promoter P_35_ and P_36_ were set as the control

In order to verify these putative promoters, the activity of the promoter was evaluated by measuring the fluorescence intensity of EGFP. The strength of the 28 promoters is shown in Fig. 3. The strength of each promoter was similar at different incubation times. Nine promoters P_5_, P_10_, P_13_, P_15_, P_18_, P_27_, P_29_, P_32_, P_34_ showed high activities across all of the ones tested at 28 °C. Compared with P_35_, their activities increased from 16-fold to 274-fold. Compared with P_36_, their activities increased from 0.5-fold to 24-fold. Moreover, these nine promoters also had high activities at 37 °C. Among the nine promoters, the activity of the P_29_ was the highest under two tested temperatures. Contrary to the RNA-seq data, P_3_, P_8_, P_9_, P_16_, P_17_, P_28_ were weak in the two tested temperatures. Since the sequences between the highly transcribed genes corresponding to P_3_, P_8_, P_9_, P_16_ and the adjacent upstream genes were very short (< 60 bp), the intergenic sequence with the appropriate length closest to the high-expressed gene was selected. It is possible that several adjacent genes may be involved in the common operon structures, and these highly expressed genes may be controlled by only one promoter in front of the entire operon. Although the sequences of the P_17_ and P_28_ are the upstream regions of the highly expressed genes, it is possible that these two genes are also involved in a common operon structure with adjacent genes. Therefore, we speculated that P_3_, P_8_, P_9_, P_16_, P_17_, P_28_ only represent intergenic sequences, not promoter sequences.

Reporter fluorescence reached its maximum at different times under the control of nine strong promoters. Under the control of P_10_, P_29_, P_32_ at 28 °C, the fluorescence intensity of EGFP reached the maximum at 24 or 36 h and then dropped. However, the fluorescence intensity controlled by the remaining six promoters (P_5_, P_13_, P_15_, P_18_, P_27_, P_34_) increased continuously at 28 °C (Fig. 3A). Interestingly, the fluorescence intensity controlled by these nine promoters all peaked at 24 h and then diminished over time to a stable level at 37 °C (Fig. 3B). Temporal changes in promoter strength can be an important factor in guiding their application for expression. Six promoters (P_5_, P_13_, P_15_, P_18_, P_27_, P_34_) may be useful for applications where late expression of a protein is required, such as the generation of metabolic products that are inhibitory to the growth of Lm at 28 °C. Therefore, these nine strong promoters can be used for heterologous antigens that need to be expressed at different stages.

### Conservative analysis of promoter region sequences

BLAST alignment was used to confirm the conservation of the nine strong promoter region sequences. The results were screened with 100 % coverage and identity. The final results showed that nine promoter regions P_5_, P_10_, P_13_, P_15_, P_18_, P_27_, P_29_, P_32_, and P_34_ existed in 82, 125, 63, 139, 28, 53, 80, 158, and 29 Lm strains, respectively. Furthermore, P_15_ existed in six *Listeria innocua* strains. Therefore, these nine promoter region sequences have varying degrees of conservation in Lm.

### Observation of fluorescence in Lm

**Fig. 4:**
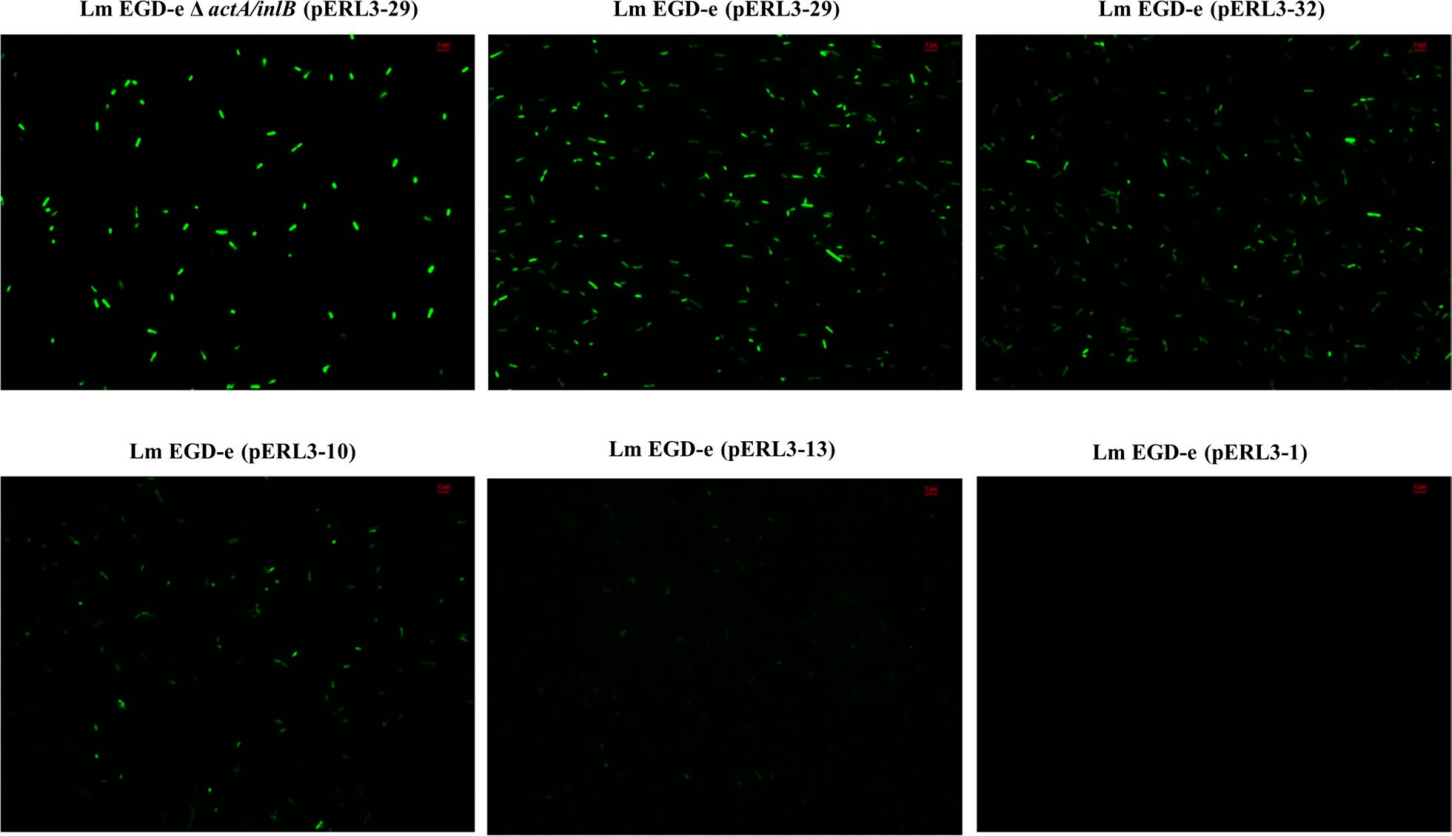
Visualization of EGFP expression in Lm at 28 °C under fluorescence microscope

Lm EGD-e and Lm EGD-e △*actA/inlB* carrying different constructed plasmids were used to observe the fluorescence intensity of bacteria under a fluorescence microscope to further verify the strength of the 28 promoters. In order to determine the potential of the use of the promoter in attenuated Lm, the constructed plasmid with the most active promoter P_29_ was electrotransformed into Lm EGD-e △*actA/inlB* to observe the bacterial fluorescence. The fluorescence intensity of Lm with different constructed plasmids varied at 28 °C. We selected several representative strains according to the fluorescence intensity of bacteria (Fig. 4). The highest fluorescence intensity of Lm EGD-e (pERL3-29) at 28 °C indicated the highest overall activity of the P_29_ (Fig. 4). Moreover, P_29_ also had high activity in attenuated Lm (Fig. 4), which will be applied to control the expression of target proteins in attenuated Lm to construct the Lm live vector vaccine. Strong green fluorescence was observed during the process of bacteria division, indicating that pERL3-29 could stably exist in Lm EGD-e and continue to express EGFP. Similarly, these results were observed in Lm cultured at 37 °C (Additional file 1: Figure S1), which illustrated that P_29_ is a stable and strong promoter. The fluorescence intensity of Lm EGD-e strains corresponding to P_32_, P_10_, and P_13_ decreased due to the different intensities of the promoters (Fig. 4). The activity of P_1_ was low so that the fluorescence of Lm EGD-e (pERL3-1) could not be observed (Fig. 4). These results demonstrated that P_29_ is the most active promoter in Lm at 28 °C. A suitable growth temperature for carps and zebrafish is 28 °C [21, 22]. Therefore, it has great potential to be used in aquatic vaccines based on Lm live vector to express target antigens.

### P_29_ controlled EGFP expression in ZF4 cells

**Fig. 5:**
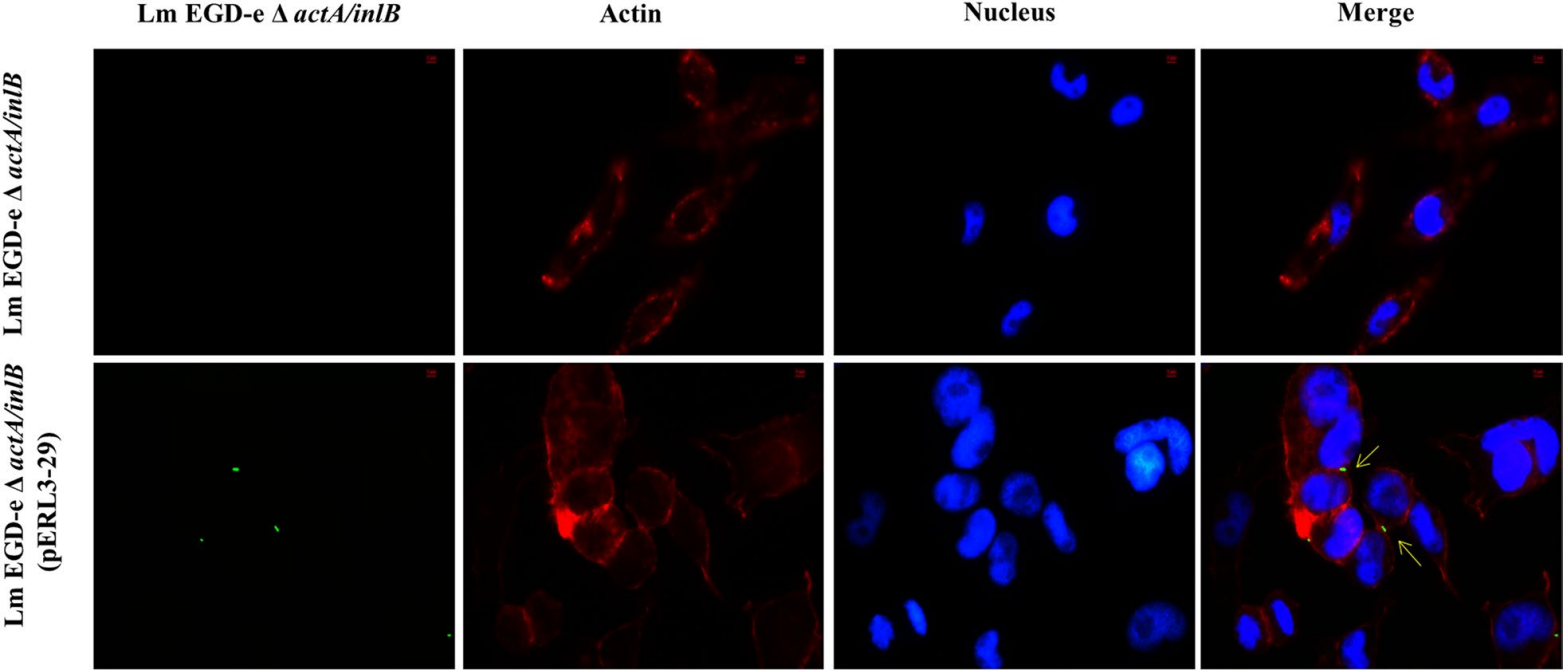
Stained images of ZF4 cells after invasion. Lm strains (EGD-e △*actA/inlB*) carrying pERL3-29 were green and ZF4 cells were stained with Acti-stain iFluorTM 555 phalloidin (red) and the nucleus were stained with DAPI (blue). ZF4 cells invaded by Lm EGD-e △ *actA/inlB* were set as control

Fluorescence staining was used to observe the expression of the EGFP in ZF4 cells in order to further explore the potential of applying P_29_ to initiate the expression of exogenous antigens in Lm. Gentamicin was added to cells for the purpose of killing extracellular bacteria. Therefore, only infectious bacteria could be observed in ZF4 cells (Fig. 5). We found that green fluorescent Lm EGD-e △*actA/inlB* (pERL3-29) invaded ZF4 cells and existed in the intercellular substance. In contrast, Lm EGD-e △*actA/inlB* was not observed in the control group because of its lack of fluorescence. These results confirmed that P_29_ could initiate EGFP expression, and had high activities in ZF4 cells. Moreover, Lm EGD-e △*actA/inlB* (pERL3-29) displayed whole-body green fluorescence so that it can also be used for its tracer in ZF4 cells. In the future, P_29_ can be used for expression of heterologous antigen and EGFP fusion proteins in attenuated Lm to detect the expression of antigen in ZF4 cells. Rahman et al. have developed a click beetle luciferase (CBR-*luc*) reporter system under the control of the P_36_ in Lm. They used the CBR-*luc* labeled Lm to invade human adenocarcinoma cell line to detect Lm in cell culture [25]. Therefore, the EGFP reporter system constructed in this study may also have the potential to evaluate infection with Lm in other cell lines. Furthermore, the P_36_ has also been used to construct luciferase-labeled Lm to detect the growth of Lm in foods, including hot dogs and Camembert [15]. The activity of promoter P_29_ was significantly higher than that of P_36_ (Fig. 3). Therefore, P_29_ may have significant applications in the construction of luciferase-labeled Lm to monitor Lm in food matrices.

### P_29_ controlled EGFP expression in zebrafish embryos

**Fig. 6:**
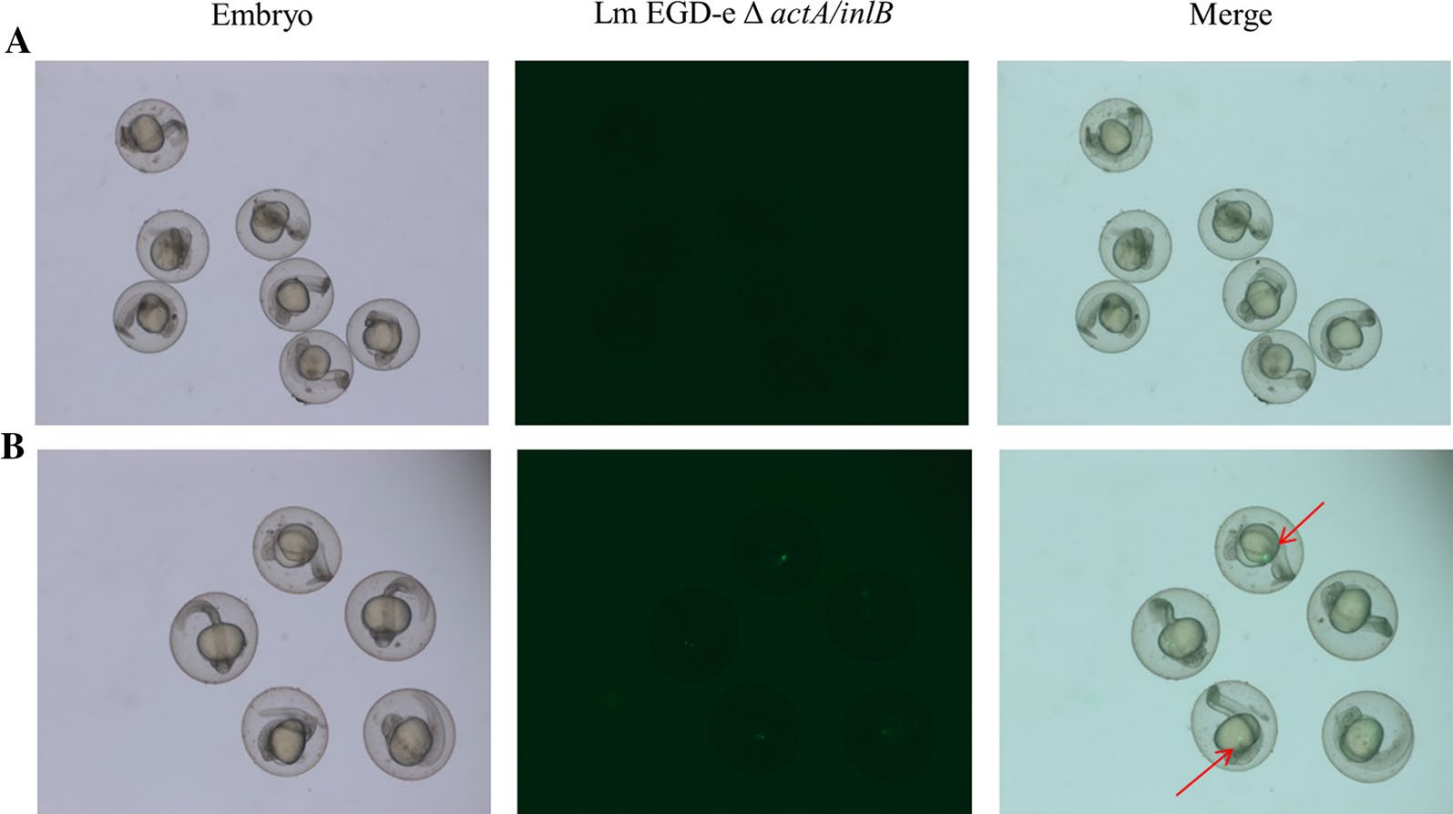
Visualization of EGFP expression controlled by P_29_ in zebrafish embryos infected by Lm EGD-e △*actA/inlB* under fluorescence microscope. **A** Lm EGD-e △*actA/inlB* (control); **B** Lm EGD-e △*actA/inlB* (pERL3-29). The zebrafish fertilized eggs were microinjected with bacteria and observed after 26 h. EGFP fluorescence was detected under fluorescence microscope

The key of attenuated Lm as a live vector vaccine is whether a promoter can initiate target gene transcription in vivo. In order to test the transcription of P_29_ in vivo, a transparent zebrafish embryo was used as a model. Each zebrafish fertilized egg was microinjected with Lm EGD-e △*actA/inlB* or Lm EGD-e △*actA/inlB* (pERL3-29) of 3000 CFU, and the expression of EGFP was observed with electromotive fluorescence zoom microscope 26 h later. As shown in Fig. 6, the zebrafish fertilized eggs with microinjection of Lm EGD-e △*actA/inlB* (pERL3-29) produced fluorescent signals at 26 h after infection, while no fluorescent signals were observed in the fertilized eggs injected with Lm EGD-e △*actA/inlB.* The results showed that P_29_ had high activities in zebrafish embryos. In our previous studies, we found that P_35_ was not sufficient to satisfy the expression level of foreign antigens in zebrafish, and to trigger a specific immune response to foreign antigens [14]. Therefore, the promoter P_29_ is expected to increase the antigen expression level of Lm live vector aquatic vaccine in zebrafish. The similar fluorescent reporter system has been used to investigate the systemic infection of Lm in vivo in a mouse model [25]. The degree of Lm infection was evaluated via the biological fluorescence signal of different organs in mice. The promoter P_29_ also has high activity at 37 °C as shown in Fig. 3. Thus, the EGFP fluorescent reporter system we constructed may be used to study the systemic infection of Lm. Furthermore, the luciferase reporter system under the control of the P_36_ has been used to track Lm in mouse mammary cancer tumor models [15]. Therefore, the promoter P_29_ is also expected to be used to construct luciferase reporter system to track Lm in mouse tumor models. It may have significant applications in the live vector vaccines based on attenuated Lm.

## Conclusions

In conclusion, we identified 26 candidate promoter regions from Lm EGD-e by RNA-sEq. Among these promoters, 9 native promoters showed great strengths according to the fluorescence intensity of EGFP at 28 °C. The strength of P_29_ is 24-fold higher than the known strong promoter P_36_. On the one hand, P_29_ had high activities in ZF4 cells. Lm can be observed directly under a fluorescence microscope without using immunological methods because of their full-body fluorescence. On the other hand, zebrafish is one of the important model vertebrates and the embryos are transparent, which makes it easy for fluorescence observation. P_29_ had been proved to initiate EGFP expression in zebrafish embryos. Therefore, Lm can also be used to study how it causes immune response in zebrafish model, which lays a good foundation for the application of attenuated Lm live vector vaccine in aquaculture. Furthermore, these 9 native promoters have high activities at 28 and 37 °C. Therefore, they could potentially be used to express a large number of target antigens in Lm, such as aquatic pathogen antigens and tumor antigens, to construct attenuated Lm live vector vaccines. In addition, the strong native promoters we identified can serve as templates for mutating their key regulatory sequences, such as the UP elements, − 35 sequences, and − 10 sequences, to build a library of synthetic promoters with higher intensity [26, 27]. Furthermore, these nine promoter regions have varying degrees of conservation in Lm. As a whole, we characterized a panel of promoters in Lm by RNA-sEq. These promoters can be used for fine-tuning the expression of various proteins in Lm. To our knowledge, this is the first well-characterized promoter library in Lm EGD-e. Our study provides a general method for the discovery and characterization of different strengths of promoters under target conditions.

## Materials and methods

### Strains, plasmids, media and the cell line

Lm EGD-e was grown in Brain Heart Infusion Broth (BHI) medium at 37 or 28 °C. *Escherichia coli* DH5α competent cell used for molecular cloning and plasmid propagation was grown in LB broth (LB) medium at 37 °C. According to the resistance marker on the plasmid, erythromycin and kanamycin antibiotics with final concentrations of 5 µg/mL and 50 µg/mL were added to the BHI and LB medium, respectively. All the strains and plasmids used in this study were listed in Additional file 1: Table S1. Cell line ZF4 was cultured at 28 °C with 5 % CO_2_, in DMEM containing 10 % FBS.

### Lm EGD-e RNA extraction

Overnight Lm EGD-e cultures were inoculated (1:100, v/v) into fresh BHI medium at both 28 and 37 °C with constant shaking (180 rpm), and 200 µL cultures were measured with a SpectraMax M2 plate reader until OD_600_ nm = 0.4. Total RNA was extracted according to the following method [17]. Briefly, 1 mL of the phenol-ethanol mixture (phenol: ethanol = 1:9, v/v) was added to 5 mL of bacterial solution, and put in an ice bath for 30 min. The bacteria were harvested by centrifugation at 4 °C (6000 rpm for 5 min), resuspended in 50 µL of 250 U/mL mutanolysin (Sigma, USA) and 50 µL of 25 mg/mL lysozyme (Sigma, USA), and the suspension was incubated at 37 °C for 30 min. Subsequently, 1 mL of Trizol was added to the reaction system and centrifuged at 4 °C (7200 rpm for 3.5 min), the collected supernatant was transferred to a new centrifuge tube for further extraction. Chloroform (200 µL) was added to the centrifuge tube, and incubated at room temperature for 5 min after shaking vigorously for 15 s. After centrifugation at 4 °C (12,000*g* for 15 min), the uppermost transparent water layer was collected and transferred to a new centrifuge tube. Then, an equal volume of isopropanol was added to the centrifuge tube, and the mixture was shaken slowly for 1 min and then left at room temperature for 10 min. After centrifugation at 4 °C (12,000*g* for 15 min), the precipitate was collected and resuspended in 1 mL of 75 % ethanol. Then, the mixture was centrifuged at 4 °C (12,000*g* for 10 min), and finally resuspended in 30 µL diethyl pyrocarbonate (DEPC) treated water.

### RNA-seq analysis

RNA quantification was performed using NanoDrop 2000c (ThermoFisher Scientific, USA). RNA integrity was checked by agarose gel electrophoresis. The RNA samples were handed over to Sangon Biotech Co., Ltd. (Shanghai, China) for transcriptome sequencing. The original image data file obtained by Illumina Hiseq™ was analyzed by CASAVA base calling and converted into original sequenced reads. Trimmomatic was used for data quality control. Transcripts Per Million (TPM) is to measure the proportion of a certain transcript in the RNA pool. TPM can reflect the strength of the promoter, and its calculation formula is as follows: 

$$TPM=\frac{{n}_{r }\times {read}_{l}\times {10}^{6}}{{g}_{l}\times T}$$

$$T=\sum _{g=i}^{G}{\left(\frac{{n}_{r}\times {read}_{l}}{{g}_{l}}\right)}_{i}$$

n_r_: length of gene reads; read_l_: number of reads aligned to gene exon region; g_l_: number of bases in exon region.

The transcription profiles of all 2952 genes in the genome of Lm were sorted based on TPM. The TPM value is used to rank each gene from the highest expression to the lowest expression in the sample, and the genes with high expression at both 28 and 37 °C are selected for further screening. The TPM values of the genes with high expression were shown in Table 1 (No. 1–34).

**Table 1:** Selected 36 promoter regions

Gene id	Name	No.	Description	Length (bp)	TPM (28 °C)	TPM (37 °C)
lmo1634	lmo1634	1	Bifunctional acetaldehyde-CoA/alcohol dehydrogenase	501	63189.35	50511.48
lmo2637	lmo2637	2	Hypothetical protein	447	36304.9	46430.03
lmo2459	gap	3	Glyceraldehyde-3-phosphate dehydrogenase	441	33735.74	18075.69
lmo2653	tuf	4	Elongation factor Tu	108	18432.82	22915.67
lmo1439	sod	5	Superoxide dismutase	300	11854.86	4118.03
lmo2455	eno	6	Phosphopyruvate hydratase	135	10842.54	7639.6
lmo2556	fbaA	7	Fructose-1,6-bisphosphate aldolase	168	10472.77	16785.1
lmo0045	ssb	8	Single-strand binding protein	278	10138.94	17333.82
lmo1468	lmo1468	9	Hypothetical protein	197	9262.2	21544.8
lmo1003	lmo1003	10	Phosphotransferase system enzyme I	306	9215.83	10326.79
lmo1257	lmo1257	11	Hypothetical protein	188	8371.88	9943.32
lmo2458	pgk	12	Phosphoglycerate kinase	134	7690.66	4931.69
lmo0210	ldh	13	l-lactate dehydrogenase	293	7175.22	7138.21
lmo2456	pgm	14	Phosphoglyceromutase	134	6841.77	4106.56
lmo2654	fus	15	Elongation factor G	192	6642.51	10207.12
lmo1541	lmo1541	16	Hypothetical protein	156	6364.55	11184.85
lmo2457	tpiA	17	Triosephosphate isomerase	134	6224.67	3356.2
lmo2411	lmo2411	18	Hypothetical protein	1223	5593.61	4311.64
lmo0250	rplJ	19	50 S ribosomal protein L10	247	4932.87	5083.75
lmo1364	cspL	20	Cold-shock protein	198	4849.28	7640.57
lmo2785	kat	21	Catalase	147	4298.38	3087.87
lmo1542	rplU	22	50 S ribosomal protein L21	156	4105.6	10392.33
lmo1424	lmo1424	23	Manganese transporter	112	4103.58	4108.83
lmo1847	lmo1847	24	Metal ABC transporter	842	4055.53	4457.12
lmo2612	secY	25	Preprotein translocase subunit SecY	495	4053.66	6469.35
lmo0251	rplL	26	50 S ribosomal protein L7/L12	247	4050.49	4177.99
lmo1399	lmo1399	27	Phosphodiesterase	300	3968.34	3260.33
lmo2016	cspB	28	Cold-shock protein	288	3876.38	7847.73
lmo2196	lmo2196	29	Peptide ABC transporter substrate-binding protein	623	3622.18	5925.35
lmo2638	lmo2638	30	NADH dehydrogenase	447	3531.4	3500.48
lmo2610	infA	31	Translation initiation factor IF-1	386	3275.76	5188.7
lmo0248	rplK	32	50 S ribosomal protein L11	126	3132.68	6800.59
lmo2615	rpsE	33	30 S ribosomal protein S5	426	3116.71	5192.1
lmo0582	iap	34	Invasion associated secreted endopeptidase	424	3070.24	3352.31
lmo0202	hly	35	Listeriolysin O precursor	171	–	–
P_help_	–	36	–	222	–	–

### Plasmid constructions

All primers were synthesized by Sangon Biotech Co., Ltd. (Shanghai, China). EGFP sequence was synthesized by Sangon Biotech Co., Ltd. (Shanghai, China) after codon optimization in Lm EGD-e with a tool JCat [28]. The Lm EGD-e genome was extracted with the TIANamp Bacteria DNA kit (TIANGEN, Beijing, China). The strong promoter P_36_ reported by Riedel et al. was synthesized by Sangon Biotech Co., Ltd. (Shanghai, China) as a positive control [15]. The P_36_ promoter sequence was amplified using P_36_F and P_36_R primers from PUC57-P_36_. P_35_ is the promoter region of *hly* gene and cloned from Lm EGD-e genome as a positive control. The EGFP gene was amplified using EGFPCAF and EGFPCAR primers from PUC57-EGFP. The promoter regions of 30 highly expressed genes were cloned from Lm EGD-e genome. Subsequently, these promoter regions and EGFP were inserted into plasmid pERL3 (digestion with *Bam*H I) using ClonExpress MultiS One Step Cloning kit (Vazyme, Nanjing, China) to create a series of derivatives of pERL3. The primers, descriptions and sequences of promoters were listed in Additional file 1: Tables S2, S3 and Table 1, respectively. The constructed plasmids were transformed into *Escherichia coli* DH5α competent cells, and the positive clones were selected using LB agar plates containing 50 µg/mL kanamycin. Finally, colonies were inoculated into 20 mL of LB media supplemented with 50 µg/mL kanamycin, and plasmids were isolated from the liquid culture using the BioSpin Plasmid DNA Extraction Kit (Bioer Technology Co. Ltd., Hangzhou, China). Plasmids isolated from *E. coli* DH5α were then submitted to BGI (Shanghai, China) for sequencing.

### Preparation of Lm competent cells

Two mL overnight Lm EGD-e cultures were inoculated into 198 mL fresh BHI medium containing 0.5 M sucrose at 37 °C with constant shaking (180 rpm), and 200 µL cultures were measured with a SpectraMax M2 plate reader until OD_600_ nm = 0.2. Subsequently, Penicillin at a final concentration of 10 µg/mL was added to the cultures, and the mixture was incubated at 37 °C under constant shaking of 180 rpm for 2 h. The mixture was put in an ice bath for 10 min. After centrifugation at 4 °C (5000*g* for 10 min), the bacterial cells were collected and resuspended in 20 mL of 10 % glycerin solution containing 0.5 M sucrose. Then, the mixture was centrifuged at 4 °C (5000 g for 10 min), and washed three times with 10 % glycerin solution containing 0.5 M sucrose. Finally, the the bacterial cells were resuspended in 1 mL of 10 % glycerol solution containing 0.5 M sucrose and divided into 100 µL per tube, and stored at − 80 °C. The preparation method of Lm EGD-e △*actA/inlB* competent cells was the same as that of Lm EGD-e competent cells.

### Fluorescence measurements

Recombinant plasmids were transformed into Lm EGD-e competent cells. The positive clones were selected using BHI agar plates containing 5 µg/mL erythromycin. Colonies were inoculated into 20 mL of BHI media supplemented with 5 µg/mL erythromycin and cultured at 28 °C (180 rpm) and 37 °C (180 rpm), respectively. Cells were collected by centrifugation at 12 h, 24 h, 36 h, and 48 h, respectively, washed three times with PBS, and then suspended. EGFP fluorescence (excitation at 485 nm and emission at 525 nm) and absorption at 600 nm (OD_600_) were measured in black 96 well assay plates (CORNING, USA) and ELISA plates (Jet Bio-Filtration Co., Ltd., Guangzhou, China) respectively using a SpectraMax M2 plate reader (Molecular Devices, USA).

### Conservative analysis of promoter region sequences

BLAST alignment was used to confirm the conservation of the promoter region sequences. The promoter region sequences (Additional file 1: Table S3) were input into the online website (https://blast.ncbi.nlm.nih.gov/Blast.cgi) and analyzed by clicking the BLAST button. If the coverage and identity were 100 %, the sequence was considered to exist in the strain.

### Visualization of Lm fluorescence

The activities of promoters were further evaluated by directly observing the fluorescence intensity of Lm carrying different plasmids. The constructed plasmid pERL3-29 was transformed into Lm EGD-e △*actA/inlB* competent cells. The positive clones were selected using BHI agar plates containing 5 µg/mL erythromycin. Then, after Lm EGD-e with different constructed plasmids and Lm EGD-e △*actA/inlB* (pERL3-29) were cultured for 12 h at 28 °C or 37 °C (180 rpm), the cells were collected by centrifugation (5000 rpm for 10 min), washed twice with ddH_2_O, and suspended. Ten microliters of the suspension were placed on the glass slide and the cover glass was put on it. Finally, they were imaged under Leica DM 2500 fluorescence microscope (Leica, Germany).

### The activity of P_29_ in ZF4 cells

ZF4 cells and Lm EGD-e △*actA/inlB* (pERL3-29) were co-cultured in a microscope cover glass (NEST, China) at a cell carbon dioxide incubator under 28 °C for 1 h. ZF4 cells without treatment were regarded as a control group. A volume of 0.5 mL of the gentamicin (200 µg/mL) was added to cells at 28 °C for 30 min to kill extracellular bacteria. Subsequently, the mixture was washed three times with PBS, and the cells were fixed with 0.5 mL of 4 % paraformaldehyde in PBS at room temperature for 30 min. After the mixture was washed three times with PBS, the cells were permeabilized with 0.1 % Triton X-100 in PBS for 5 min. Then, the mixture was washed three times with PBS, and the actin of the cells was stained with iFluor™ 555 phalloidin (YEASEN, Shanghai, China) at room temperature and kept in dark the place for 90 min. After the mixture was washed three times with PBS, cell nucleus were stained with DAPI (YEASEN, Shanghai, China) at room temperature and kept in the dark place for 5 min. Finally, the mixture was washed twice with PBS, and images were observed and captured on Leica DM 2500 fluorescence microscope (Leica, Germany).

### The activity of P_29_ in zebrafish embryos

Zebrafish embryos were cultured in a laboratory breeding system. Overnight cell cultures of Lm EGD-e △*actA/inlB* and Lm EGD-e △*actA/inlB* (pERL3-29) were inoculated (1:100, v/v) into 20 mL fresh BHI medium and BHI containing erythromycin respectively. They were cultured at 28 °C for 12 h. Then the cells were harvested and resuspended in PBS. When the OD_600_ nm of 200 µL cell cultures was 1.2, the concentration of the bacterial solution was 1 × 10^10^ CFU/mL. The concentration of the bacterial solution was adjusted to 3 × 10^9^ CFU/mL by PBS, and the volume of the microinjection was 1 nL. Zebrafish fertilized eggs at the age of 1 h were selected and each of them was microinjected with 3000 CFU bacteria. Then infected fertilized eggs were maintained in fresh water. The EGFP expression in vivo in zebrafish embryos was finally analyzed with an electromotive fluorescence zoom microscope (AXIO zoom V16, ZEISS, Germany) at 26 h after infection.

## Supplementary Information

**Additional file 1: Figure S1**. Visualization of EGFP expression in Lm at 37 °C under fluorescence microscope. **Table S1**. Strains and plasmids used in this work. **Table S2**. Primers used in this study. **Table S3**. Sequences of promoters used in this study.

## Data Availability

All data generated or analysed during this study are included in this published article.
