# Supplementary material for: Systematic identification of a panel of strong promoter regions from Listeria monocytogenes for fine-tuning gene expression
Source: Microb Cell Fact. 2021 Jul 12;20:132. doi: 10.1186/s12934-021-01628-w (PMC8273982; doi:10.1186/s12934-021-01628-w)
Supplement: Supplementary file 1 — Additional file 1: Figure S1. Visualization of EGFP expression in Lm at 37 °C under fluorescence microscope. Table S1. Strains and plasmids used in this work. Table S2. Primers used in this study. Table S3. Sequences of promoters used in this study. [file 12934_2021_1628_MOESM1_ESM.docx]

**Additional file 1**

**Systematic Identification of a Panel of Strong Promoter Regions from *Listeria monocytogenes*** **for** **Fine-Tuning Gene Expression**

Qianyu Ji^1^, Junfei Ma^1^, Shuying Wang^1^, Qing Liu ^1^*

^1^School of Medical Instrument and Food Engineering, University of Shanghai for Science and Technology, Shanghai 200093, PR China

* Corresponding author at: School of Medical Instrument and Food Engineering, University of Shanghai for Science and Technology, Shanghai 200093, PR China.

Tel.: +86 021 65710369

E-mail address: liuq@usst.edu.cn (Q. Liu)

Table S1. Strains and plasmids used in this work

| Strains and plasmids | Description | Sources |
| --- | --- | --- |
| *Listeria monocytogenes* EGD-e | Wild type | a gift from Professor Qin Luo of  Central China Normal University |
| *Listeria monocytogenes* EGD-e △*actA/inlB* | Double-deleted Lm strain | Our lab |
| *Escherichia coli* DH5α competent cell | Host for cloning | Purchased by Vazyme, Nanjing, China |
| PUC57-P_36_ | #36 promoter cloning vector | Purchased by Sangon  Biotech Co., Ltd. (Shanghai, China) |
| PUC57-EGFP | EGFP cloning vector | Purchased by Sangon  Biotech Co., Ltd. (Shanghai, China) |
| pERL3 | *Listeria monocytogenes* and *Escherichia coli* shuttle vector, Em^r^ and Kan^r^ | a gift from Professor Qin Luo of  Central China Normal University |
| pERL3-1 | pERL3 derived, carrying #1 promoter and a EGFP gene | This study |
| pERL3-2 | pERL3 derived, carrying #2 promoter and a EGFP gene | This study |
| pERL3-3 | pERL3 derived, carrying #3 promoter and a EGFP gene | This study |
| pERL3-4 | pERL3 derived, carrying #4 promoter and a EGFP gene | This study |
| pERL3-5 | pERL3 derived, carrying #5 promoter and a EGFP gene | This study |
| pERL3-6 | pERL3 derived, carrying #6 promoter and a EGFP gene | This study |
| pERL3-7 | pERL3 derived, carrying #7 promoter and a EGFP gene | This study |
| pERL3-8 | pERL3 derived, carrying #8 promoter and a EGFP gene | This study |
| pERL3-9 | pERL3 derived, carrying #9 promoter and a EGFP gene | This study |
| pERL3-10 | pERL3 derived, carrying #10 promoter and a EGFP gene | This study |
| pERL3-11 | pERL3 derived, carrying #11 promoter and a EGFP gene | This study |
| pERL3-13 | pERL3 derived, carrying #13 promoter and a EGFP gene | This study |
| pERL3-15 | pERL3 derived, carrying #15 promoter and a EGFP gene | This study |
| pERL3-16 | pERL3 derived, carrying #16 promoter and a EGFP gene | This study |
| pERL3-17 | pERL3 derived, carrying #17 promoter and a EGFP gene | This study |
| pERL3-18 | pERL3 derived, carrying #18 promoter and a EGFP gene | This study |
| pERL3-19 | pERL3 derived, carrying #19 promoter and a EGFP gene | This study |
| pERL3-20 | pERL3 derived, carrying #20 promoter and a EGFP gene | This study |
| pERL3-24 | pERL3 derived, carrying #24 promoter and a EGFP gene | This study |
| pERL3-25 | pERL3 derived, carrying #25 promoter and a EGFP gene | This study |
| pERL3-27 | pERL3 derived, carrying #27 promoter and a EGFP gene | This study |
| pERL3-28 | pERL3 derived, carrying #28 promoter and a EGFP gene | This study |
| pERL3-29 | pERL3 derived, carrying #29 promoter and a EGFP gene | This study |
| pERL3-31 | pERL3 derived, carrying #31 promoter and a EGFP gene | This study |
| pERL3-32 | pERL3 derived, carrying #32 promoter and a EGFP gene | This study |
| pERL3-34 | pERL3 derived, carrying #34 promoter and a EGFP gene | This study |
| pERL3-35 | pERL3 derived, carrying #35 promoter and a EGFP gene | This study |
| pERL3-36 | pERL3 derived, carrying #36 promoter and a EGFP gene | This study |

Table S2. Primers used in this study

| Name | Primer sequence (5’→3′) |
| --- | --- |
| P_lmo1634_F | CCCCGGAATTCCCGGGGATCCTTTTTACTTGTGTTTATCTT |
| P_lmo1634_R | ACCTTTAGAAACCATTTTCCAGAACCTCCTAATAT |
| P_lmo2637_F | CCCCGGAATTCCCGGGGATCCATGCTTCCTCAACTCCTTGT |
| P_lmo2637_R | ACCTTTAGAAACCATGTTTTGCTCCCACCTTTTGT |
| P_lmo2459_F | CCCCGGAATTCCCGGGGATCCAAGACGTATTTTGTCTCACG |
| P_lmo2459_R | ACCTTTAGAAACCATTTTTAATTGCTCCTCATCTT |
| P_lmo2653_F | CCCCGGAATTCCCGGGGATCCTTGATTTTTTTCGCAACATC |
| P_lmo2653_R | ACCTTTAGAAACCATTTTAAAATATCCTCCTCGAT |
| P_lmo1439_F | CCCCGGAATTCCCGGGGATCCTCTTTTTTCAACACCTCG |
| P_lmo1439_R | ACCTTTAGAAACCATTAAAAATTCCTCCTTGTATT |
| P_lmo2455_F | CCCCGGAATTCCCGGGGATCCTTTTAGTTATTACTTTAGTT |
| P_lmo2455_R | ACCTTTAGAAACCATTTATAATTCTCTCCTTTGTT |
| P_lmo2556_F | CCCCGGAATTCCCGGGGATCCAGCGTTTACACCAGAAAATT |
| P_lmo2556_R | ACCTTTAGAAACCATAATACAATTTCCTCCCTCAG |
| P_lmo0045_F | CCCCGGAATTCCCGGGGATCCGCTTTGAAAAATGAGAATCC |
| P_lmo0045_R | ACCTTTAGAAACCATCTACTCTACACCTCCTTGTG |
| P_lmo1468_F | CCCCGGAATTCCCGGGGATCCCATTTCAAAGTTTTATACGA |
| P_lmo1468_R | ACCTTTAGAAACCATCTCTCTTTCCCTCCCTC |
| P_lmo1003_F | CCCCGGAATTCCCGGGGATCCTTTTGAACCTTCCTTAAATT |
| P_lmo1003_R | ACCTTTAGAAACCATAATTTACATTCTCCTTTGTA |
| P_lmo1257_F | CCCCGGAATTCCCGGGGATCCGTGTCTCCCTCCCATTTCAT |
| P_lmo1257_R | ACCTTTAGAAACCATGTGTGTCATCTCCTTATGGG |
| P_lmo2458_F | CCCCGGAATTCCCGGGGATCCTTAGCAGATTAATTTTACTG |
| P_lmo2458_R | ACCTTTAGAAACCATTTAGAGTTCCTCCATTAATT |
| P_lmo0210_F | CCCCGGAATTCCCGGGGATCCAAAAAATCACTCCACTCATT |
| P_lmo0210_R | ACCTTTAGAAACCATTTCGAATTCCTCCTATAAAA |
| P_lmo2456_F | CCCCGGAATTCCCGGGGATCCTTAGCAGATTAATTTTACTG |
| P_lmo2456_R | ACCTTTAGAAACCATTTAGAGTTCCTCCATTAATT |
| P_lmo2654_F | CCCCGGAATTCCCGGGGATCCTTAACTAAATTTCAAGTAAC |
| P_lmo2654_R | ACCTTTAGAAACCATGGGATAAATTCCTCCTTC |
| P_lmo1541_F | CCCCGGAATTCCCGGGGATCCAATCAACAATTGGTTGACAT |
| P_lmo1541_R | ACCTTTAGAAACCATACTTGCACCTCCTCATATAC |
| P_lmo2457_F | CCCCGGAATTCCCGGGGATCCTTAGCAGATTAATTTTACTG |
| P_lmo2457_R | ACCTTTAGAAACCATTTAGAGTTCCTCCATTAATT |
| P_lmo2411_F | CCCCGGAATTCCCGGGGATCCATGATTGATATCCAAAAAAT |
| P_lmo2411_R | ACCTTTAGAAACCATAATCCAAAATACTCCTTTGT |
| P_lmo0250_F | CCCCGGAATTCCCGGGGATCCAATTTAACTTGACCAGCCCC |
| P_lmo0250_R | ACCTTTAGAAACCATTTTTCCACCTCCGTCCAACA |
| P_lmo1364_F | CCCCGGAATTCCCGGGGATCCTCATAATTATCTAGTAATTTC |
| P_lmo1364_R | ACCTTTAGAAACCATGTTCATGTTCCTCCTC |
| P_lmo2785_F | CCCCGGAATTCCCGGGGATCCATGATAAAAAGCAATCTCTC |
| P_lmo2785_R | ACCTTTAGAAACCATATGTATATACCTCCATAACA |
| P_lmo1542_F | CCCCGGAATTCCCGGGGATCCAATCAACAATTGGTTGACAT |
| P_lmo1542_R | ACCTTTAGAAACCATACTTGCACCTCCTCATATAC |
| P_lmo1424_F | CCCCGGAATTCCCGGGGATCCAAATATGCGAAAACACAACC |
| P_lmo1424_R | ACCTTTAGAAACCATGCTCCCTGACCTTCTTTCAT |
| P_lmo1847_F | CCCCGGAATTCCCGGGGATCCGAGTTGTTGTTTTTAGAAGG |
| P_lmo1847_R | ACCTTTAGAAACCATTCGATAACCTCCCTTTTTTT |
| P_lmo2612_F | CCCCGGAATTCCCGGGGATCCGCTTAAAATTATTATGCAAT |
| P_lmo2612_R | ACCTTTAGAAACCATTAGATCACCTCAGTTTTTCC |
| P_lmo0251_F | CCCCGGAATTCCCGGGGATCCAATTTAACTTGACCAGCCCC |
| P_lmo0251_R | ACCTTTAGAAACCATTTTTCCACCTCCGTCCAACA |
| P_lmo1399_F | CCCCGGAATTCCCGGGGATCCATAACACTAAATACGTGATA |
| P_lmo1399_R | ACCTTTAGAAACCATCCTTACACCTCCTTTGCTA |
| P_lmo2016_F | CCCCGGAATTCCCGGGGATCCTATAAAAACCCTGTTATAAG |
| P_lmo2016_R | ACCTTTAGAAACCATATTTCACAAACCTCCAAAAA |
| P_lmo2196_F | CCCCGGAATTCCCGGGGATCCTTCTTCACCTCTAATTAAAA |
| P_lmo2196_R | ACCTTTAGAAACCATTAAGTAGACCTCCCTTTTTT |
| P_lmo2638_F | CCCCGGAATTCCCGGGGATCCGTTTTGCTCCCACCTTTTGT |
| P_lmo2638_R | ACCTTTAGAAACCATATGCTTCCTCAACTCCTTGT |
| P_lmo2610_F | CCCCGGAATTCCCGGGGATCCGGAGTGAGCTGTGCTGAATC |
| P_lmo2610_R | ACCTTTAGAAACCATATGTTTGCTACCTCCTTCTT |
| P_lmo0248_F | CCCCGGAATTCCCGGGGATCCAAAAATCTTGCAACTATACA |
| P_lmo0248_R | ACCTTTAGAAACCATGAGACATACCTCCTTAAGTC |
| P_lmo2615_F | CCCCGGAATTCCCGGGGATCCTAATGTTAGTTCCTAAACGT |
| P_lmo2615_R | ACCTTTAGAAACCATTTGCTTCACCACCAATTTCT |
| P_lmo0582_F | CCCCGGAATTCCCGGGGATCCTATATAACATCCTCCATACC |
| P_lmo0582_R | ACCTTTAGAAACCATATTCATAAAACTCCTCTCTT |
| EGFPCAF | ATGGTTTCTAAAGGTGAAGA |
| EGFPCAR | TGGCTGCAGGTCGACGGATCCTTAATGATGATGATGATGAT |
| pERL3F | TCGGAATCGTTTTCCGGGAC |
| pERL3R | CTCTGTCGCTATCTGTTGCG |
| P_lmo0202_F | CCCCGGAATTCCCGGGGATCCCGACGATAAAGGGACAGCAG |
| P_lmo0202_R | ACCTTTAGAAACCATCATGGGTTTCACTCTCCTTC |
| P_36_F | CCCCGGAATTCCCGGGGATCCGATCTCGAGATCTGCAGGAT |
| P_36_R | ACCTTTAGAAACCATCATGGGTTTCACTCTCCTTC |

GGATCC sequence is the restriction site for *Bam*H I.

Table S3. Sequences of promoter regions used in this study

| ID | Promoter | Sequence 5’→3’ |
| --- | --- | --- |
| lmo1634 | 1 | TTTTTACTTGTGTTTATCTTACATAAGAAAATCACGAAAGTCAAGAAAATAAAACTACTTTATACTAAAAAGGTTTCAAAACGATAATTAATAAACAAGTAAGAAAAAAGAACTACCCATCTAAAATATATTTCACTTCAGAAAAAGCATGCTATTCTCTCTATTATAATGAACACTAACTTCCTTAAAAACCCTATAAAATATATGTTTTCAAATGATAATTCGCTTAAATCCCAATGATAAAAACACTCATATAAACTATGCCTAATTAACTATAATACAACTTTACAAAAGGACTTTTCAACCATCTTTATCAATTTGTGAAGTTTTTCACGTGAAACACTGGACAAACTTTTATTGATGGATTATACTAACGGTGTAATCAAATAAAACAAAAGAATACGGCAAAGCAACAAGCTAAATTTTTTTGGATTATCAGGGACGTCAAAAGTCTACTATGGACGTTTTGAGTCCCGAACAAATATTAGGAGGTTCTGGAAA |
| lmo2637 | 2 | ATGCTTCCTCAACTCCTTGTAAGTTTATTCACAAAAATAAAATGAGTCCAAAATTCCCCACAGTATTATTATAACCTATCATAGTTTAACAGAAACCTCCTACTAATGTCTAGATAGTAAAGAACAATTCTAATCTAAATTTGAAAAATAGGACTTTGTGCAAATGTTACATAATTAAGCAGGCTAAAAGTCCTTGTTTAACATAGGATGTTAGACATTTTTGTTTGCTATTTTGCTAGAAAAGGTTGGTTTAAGCCATCTTTTGTATATCAATAGTCATTATTTTGAAAATTAGATGAATTATGTCTTTAGAATTACAGTAGACAAAACCGGGCGCTGATGGTATCATTTGTATTGTAAAAGTGATATCGCTTTCGCAGACTATCGGTTTGTGTTCACGAAATCACAAACAAAGTTAATCAATAAAACAAAAGGTGGGAGCAAAAC |
| lmo2459 | 3 | AAGACGTATTTTGTCTCACGAGCGGTATGTGGGACAAAATACGTCTTTTTAAAACAAAAAAATCACTAAGAGCATATTTCATTTTTTAGAGAGGTGGAAAACAACTAGTGTAGCCTATTTAGAAGTTTGCAGTTTAAGAATTAATTGTAAATTTCAGGTGAATATTTCATAAATGTCCCACTTAACACTTGAAGATTTGTGCCAAGACTGTTAGAATAGGGAACGTAGGGTAGTTAAAGAACATAAAAAAAGATTCACCAGCGTAAGCAGAAAGAGAAGGGGTAAAAATCACATTGAGAGACACTTTGTTAACAAGCTGAAGTAGCTAGTTTAATAGTCTTGAAAGTTGTAAAAGCCTCTATCTCTACTTTTTTTAGTTCTAACGGGACGTCAGATGACTAAGTGGGTCATTAAGCGTCCAAAGATGAGGAGCAATTAAAA |
| lmo2653 | 4 | TTGATTTTTTTCGCAACATCAAGTATAACTTTAGTTAGAAGTATTACTTAGTTTAAATTTAAGCTAAGTAAAAAATAATTATCGAATTATCGAGGAGGATATTTTAAA |
| lmo1439 | 5 | TCTTTTTTCAACACCTCGCCTAATGGTTTAACTTTTGAGTTTCAGGGAAAAAGATACATGTATGTGCAAAAGAGCGTACTTTTCCACCTAAACTTAATCATAGCTTGAAATGGTCGCAAAATCAATGTTGAAACATCAATGAGCAGAAAAATTGCAAGCATTTCGGGAGCATGGTAGGCTAAATGGTGTAAGAAGAAACTGTTTTTAAGGTTGATAGTAGTTCTATTGAAATAGGACATGAAACTTTTGCCTTATACGTCATTTCTTTTCACGTAAAAACAATACAAGGAGGAATTTTTA |
| lmo2455 | 6 | TTTTAGTTATTACTTTAGTTTTTTTGCAAACAGAGCTACAATAAATACGAATTTAAAAACGGATGGGTCTTTGAATTTGAAGTGAATATTATGTTCATTTCAATCAATTAATCCCAACAAAGGAGAGAATTATAA |
| lmo2556 | 7 | AGCGTTTACACCAGAAAATTTTCTAGTTTTTATGGTAAATTCATCACAAATCTGTTATCATATGTTTGTTAGGGGAAAATAGCTGCCACTATTTTTCACGGCGGAAATTCAAATCTTAGGGGAAATCAAAAATGGTTTTCCTAAATTCCTGAGGGAGGAAATTGTATT |
| lmo0045 | 8 | GCTTTGAAAAATGAGAATCCCCTTTCAAAATGCGACTTTCGCTAAAAGTATTGTTGGCGATTTTAAGATGTGCTATAGTGTTTTCAAACCAGAATTTTCGAGAAATATCTTGCTGGTTAGTTTGCGCTATGATATAATATTTTATTGTGAGTAATTATAAATTATTCCTTGCTCGCTCTTGATTTTAAGGCTTGCTAAAGGCAGTCTGGATTTCAGGGATATGGTATGGTTTCATGCTATATCAGAGCCGCTTAAGACCACAAGGAGGTGTAGAGTAG |
| lmo1468 | 9 | CATTTCAAAGTTTTATACGAAAGTTAACGATTTCATGTTGACGATGCCCGATGTTTCGTATATAATTTTTAATGAGTGAATGCTTATCTGCATTACTTCAGAATAATTCATAATTTATTTTCCGTTATGTCTTATTCTGGAACTTAAATGAAGGATTTAAGTGAGTGCATGTAAGTGCCGGAGGGAGGGAAAGAGAG |
| lmo1003 | 10 | TTTTGAACCTTCCTTAAATTTTGCACTAAAAAACTATTAAAATCAACTATAAGCCATTAAAAAGTGCTATTTAATTCAAAACAGAAACAATTTAGTGATAAATTAAAAAAAACGCTCTATTTTTTCTATTTAAAAAGAGCGAAATAAGGTGCTCTACAAAAAAACATTCTAAAAAATTTTTAATCAAATAGTTGTAACAATAGGTAAAACATGGTACTCTTTTTAATTGAGGGATAGTCTTTTTGGCTGAAATTCTTTCGGCTTAAAAGGTTACAACAAATTTCTTTACAAAGGAGAATGTAAATT |
| lmo1257 | 11 | GTGTCTCCCTCCCATTTCATCTTAATATTAGTATAACATCTGGTAGTTATTTTCACTATTTCACTGCCTTTTATTAGGACTTTTGTATAACTAGTTAAAAAGTTACAGTTCCAACTATTCATTTATCCCGACTTGATTGTTCACATAATCACAATGTGTTATAATAAACCCATAAGGAGATGACACAC |
| lmo2458 | 12 | TTAGCAGATTAATTTTACTGAAGTGCTAGTCACATCAGCAATAACAAGAATAAGCGGAGACACTATTGTTTCCGCTTATTTTCTGAAATAACGGGGGCGACCCCAATCCTTTAAAATTAATGGAGGAACTCTAA |
| lmo0210 | 13 | AAAAAATCACTCCACTCATTCAATTTTGGTACATCTACATTATATCTACCCTTTAGAAAAATAGCAAAACAAAGGAATAGCCTTATTTTGTTTTGTAAGGTTTATAATTCTCAATAAAAGGTAAACAGAAAATAGCGGTTACTCGCTAATGTGGACGCTTCCAAACTGTACTACAGCGTTTTCACAAATTCATTATAACAACTTTTTTAAATTTAGGACGTTCAACGCTTCACAAATGAAAAATACATGGTATACTGTACTAGTAAATAGATTTTTTATAGGAGGAATTCGAA |
| lmo2456 | 14 | TTAGCAGATTAATTTTACTGAAGTGCTAGTCACATCAGCAATAACAAGAATAAGCGGAGACACTATTGTTTCCGCTTATTTTCTGAAATAACGGGGGCGACCCCAATCCTTTAAAATTAATGGAGGAACTCTAA |
| lmo2654 | 15 | TTAACTAAATTTCAAGTAACACCTTGACATCTAAACCTCAAGGTGTTACTATATTCAAGGTGCCTCTACTATAAACAGAGGTCTGAAAATAAAACAGCGTTGTAGGTCGAACTTACAGCCATTTTATTTTACCGAAAAAATGAACCACCTGGATGTGTGGAACTACTAAATAAGGAAGGAGGAATTTATCCC |
| lmo1541 | 16 | AATCAACAATTGGTTGACATAGGCTACTTGCTTATGCTATCATTTTACTGTTATGTTTGTAGCGCACCAAGCTACAACCGCACAGAGCTGGGTCAAGTATTTACTTCGTGTAAATCCCCTGTGATGGCGAGTCTTAGTATATGAGGAGGTGCAAGT |
| lmo2457 | 17 | TTAGCAGATTAATTTTACTGAAGTGCTAGTCACATCAGCAATAACAAGAATAAGCGGAGACACTATTGTTTCCGCTTATTTTCTGAAATAACGGGGGCGACCCCAATCCTTTAAAATTAATGGAGGAACTCTAA |
| lmo2411 | 18 | ATGATTGATATCCAAAAAATTCGGGCGGATTTTCCAATTTTAGCTCAAGAAATAAATGAAAAACCGCTAGCTTATTTAGATAATGCTGCCACTTCACAAAAGCCGAAACAAGTTATTGAAGCTTTAACACATTACTATGAGTTTGATAATGCGAACGTTCACCGTGGTGTGCATACGCTTGCGGCAAGAGCGACGGATGCTTATGAATCAGCTCGAGGTAAAGTAGCCAAGTTTATTCATGCGCGTGAAGTAGCAGAAATTATTTTCACAAGAGGCACTACTTCAGCGATTAATTTAGTTGTAGATAGTTACGCGGAAGCAAATATTGAAGCTGGCGATGAGATAGTTATTTCTTATTTAGAGCATCATTCTAATTTGATTCCATGGCAACAACTAGCTAAACGCAAAGGCGCGGTTTTAAAATATATCGAACTAGAAGAAGATGGCACGATTTCAGTGGAACAAGCGAAAAAAACGATTGGCGAGAAAACGAAAATCGTTGCGCTAGCACATGTTTCTAATGTTCTAGGGACAATCACGCCAATTAAAGAAATCGCAGCAATTGCTCATCAATTTGGAGCAGTCATTCTCGTTGACGGTGCGCAAGCTGTGCCTCATATGGAAGTCGATGTAGTTGATTTAGATGCTGACTTTTATGCTTTTTCAGGGCATAAAATGATGGCTCCTACTGGCATTGGCGCTTTGTATGGCAAACGTGAATTGCTTGATGCGATGGAACCTACCGAATTTGGCGGAGAAATGATTGATTTTGTTGAATTATACGATTCGACTTGGAAAGAACTACCTTGGAAATTTGAAGCCGGAACACCGATTATTGGCGGAGCAATTGCGCTAGGTGCGGCGATTGATTACTTGGCAGAAGTCGGACTCGAAAACATTCACGCACATGAACAAGCATTAGCCAGCTACGCGATGGAAGAAATGAGCAAAATCGAAGGCATTACCATTTACGGGCCGAAAGATGCAAGTAAACGTTGTGGTTTAGTGACTTTTAATTTAGAAGGCGCACACCCACATGATATTGCGACTATTTTGGACGAAGATGGGGTGGCGATTCGAGCTGGACATCACTGTGCACAACCGTTGATGAAATGGCTGGACGTTTCTTCCACAGCTCGCGCAAGCTTTTATATTTATAATACAAAAGAAGAAATTGATGCGCTTATAGATGGCCTCAAGTTAACAAAGGAGTATTTTGGATT |
| lmo0250 | 19 | AATTTAACTTGACCAGCCCCTTGTCTTTTGATAAGATAGGCTAGTTGAAACAATCAAATATCCTTACCGTAGACAGTTGGCGCGTTTATCGCTTAAATTTGCCACCCGAGGGATTTCTTTTGAAGTGTGCGTCCGCGTGCATTTTACAAAAACCTCTGACGTCTACGGTGTCAGAGGTTTTTTGTTGCTGATCAAACGGAACTTGTGTCTGTTTAATTAGATTTAATTGTTGGACGGAGGTGGAAAA |
| lmo1364 | 20 | TCATAATTATCTAGTAATTTCAAAATTTTTTCACATATATAATTCAAATTGATTTGCTTTTCCTAAAATACCGTGTTATACTAATGTAAGATTATTTTTGTGGGTGAAAGATACGATTGTGAACAACTTTCCATCTCGTGCCGTTAAGCAAGAATAGTAAATAATTAGTGTGCATAACACACGAGGAGGAACATGAAC |
| lmo2785 | 21 | ATGATAAAAAGCAATCTCTCCTAAGAGATTGCTTTTTATAAGCCTTTTAGTTGAAGTTTGTCTCTCCCTTCTTTAAAATATAAATAGTATCATTTAATAATTATTATAAATAAAAATGATATAGAATTGTTATGGAGGTATATACAT |
| lmo1542 | 22 | AATCAACAATTGGTTGACATAGGCTACTTGCTTATGCTATCATTTTACTGTTATGTTTGTAGCGCACCAAGCTACAACCGCACAGAGCTGGGTCAAGTATTTACTTCGTGTAAATCCCCTGTGATGGCGAGTCTTAGTATATGAGGAGGTGCAAGT |
| lmo1424 | 23 | AAATATGCGAAAACACAACCTATCATTTATGCATAAAGGTTGTGTTTTTCGTTTGACAAAGAATGTTTCCCGTAGTAAACTTTTTATCATAAATGAAAGAAGGTCAGGGAGC |
| lmo1847 | 24 | GAGTTGTTGTTTTTAGAAGGTTTAATGCAATATAGTTTTTTACAAAAAGCCCTTATTACTTCTGTGACGGTTGGTATTGTTTCAGGTGTTATTGGTAGTTTTATTATTTTACGAGGTATGTCGCTTATGGGGGATGCGATTTCTCATGCAGTGCTTCCAGGAGTGGCGATTTCTTATATGATGGGGATGAACTTCTTTATTGGTGCAGCTACATTCGGCATCGCGGCGGCACTTGGAATCGGTTTTGTTAATCAAAAAAGTCGGATAAAAAATGATACAGCGATTGGAATTGTTTTTAGTGCATTTTTTGCACTTGGAATTATTTTAATATCCTTTGCGAAAAGTAGTACGGATTTGTATCATATTTTATTTGGAAATGTGCTTGCGGTGCGGAGTTCGGATATGTGGATGACAATTATTATTGCCATTATCGTGATTTCATTAGTAGCCCTATTTTACAAAGAGTTTCTAGTTAGTTCGTTTGATCCAGTGATGGCAGAAGCATATGGTCTCAATGTGAAATTCTTGCATTACTTCTTGATGTTACTTTTAACACTTGTAACGGTTTCCGCTTTGCAAACGGTTGGAATTATTTTAGTTGTGGCGATGTTAATTACGCCAGCTGCAACAGCTTATCTGCTTACGAATAAATTATCCAAAATGATTGTTCTTGCTTCTACTTTTGGAGCAGTGAGTGCGATTATCGGACTTTACTTTAGTTACATTTTCAACTTAGCATCTGGTGCGGCTATGGTTTTAGTTGCGACAATTATTTTCTTTATTGCCTTTTTATTCGCACCGAAACAAGGCTTGCTATTTTCTAAAAAAAGGGAGGTTATCGA |
| lmo2612 | 25 | GCTTAAAATTATTATGCAATTGATAGAATTTGTTAAATAGGAGGAGGTGCTTGACATGAAACTACATGAACTTAAGCCTTCAGAAGGTTCTCGTAAAGAACGTAATCGTGTTGGTCGTGGAACAGGCTCTGGTAACGGCAAAACTTCAGGACGCGGTCATAAAGGACAAAAAGCTCGTTCTGGTGGTGGCGTACGTTTAGGTTTTGAAGGTGGACAACTTCCACTTTTCCGTCGTATTCCAAAACGTGGATTCACAAATATCAACCGTAAAGAATTTGCTATCGTGAACTTAGATGTTTTAAACCGCTTTGAAGATGGTACAGAAGTAACACCAGAACTTTTAGTTGAAACTGGAATTATTCGTAATGAAAAATCCGGAATCAAGATTTTATCTAATGGAAATATCGAGAAAAAACTTACTGTGAAAGCGAACAAATTCTCTGCAGCTGCGAAAGAAGCAATTGAAGCAGCTGGCGGAAAAACTGAGGTGATCTA |
| lmo0251 | 26 | AATTTAACTTGACCAGCCCCTTGTCTTTTGATAAGATAGGCTAGTTGAAACAATCAAATATCCTTACCGTAGACAGTTGGCGCGTTTATCGCTTAAATTTGCCACCCGAGGGATTTCTTTTGAAGTGTGCGTCCGCGTGCATTTTACAAAAACCTCTGACGTCTACGGTGTCAGAGGTTTTTTGTTGCTGATCAAACGGAACTTGTGTCTGTTTAATTAGATTTAATTGTTGGACGGAGGTGGAAAA |
| lmo1399 | 27 | ATAACACTAAATACGTGATAGGAATTTACTTCCTGTCACGTATTTTTTACTATTTTAAAAAAACTAAGGAAAATAACCGTACATTTCATTCCTTTTCTTGACATTGTATGCTTTGACCTATAAAATTAAGTTGTATATTTTATATTGCTGGGAGACAAGGGGGAAGTTTTTTCCTTTGAAAATCGGCTTCAACTGAATGGGGAGATAAGCACTCGGTTGTTGATGAAAAATACGCATGTGCCAGAATTCTGGCCACCGACATAACAATAACGAAAATTGAATAGCAAAGGAGGTGTAAGG |
| lmo2016 | 28 | TATAAAAACCCTGTTATAAGAATTAGCTTATAGCAGGGTTTTTCAGATAGTAAACTGAATAATCTATTTTGTAACCGTACAGCTTTTTCACTTGATTTATTTGCTCGTTGGCGTTAATATATAAGTGTTGATAGTAGCTAATTTACTTATTATCAAGGATATTGATGTGAAGTGAGTATCTTTGCTAATATGTGAATTTTGTAATTGAAAAAATGATTAGCTTGTTGTTAACATCTTTTTCGTTAGTAAAAGAAACATATTAAGAAAATTTTTGGAGGTTTGTGAAAT |
| lmo2196 | 29 | TTCTTCACCTCTAATTAAAATTCATAATTCACTCTATCACAATTTGCATAGAAAGACTATATGATAGAAGGAAGCTATCATCCAATGTTAGAAATTTGTCCGTTTTTAGTAAAATATTCCGCCAAGTGTCTTTACAGATTATTAACAGAAAAATCCCTTTATATCAACGTTTTTCGAATTTATTACAAAATTTAATTAAATTTTTGATATTTTTATCTAGACAACAGCTCAAAAAAAGTATATAAATATAATAATCCTTTAAATTTTAAAGGGGTTCGATATTTTCTTTTATACAAAAAAGATAACTTCTTCCAGTTTTAACTTAATTGGCAAAGTTCATCGTAAATTTATCAATTTAAATGGTTATGTTTTCTGAAAACTTAATCAAAAGGTTTAGCAAAGTATACATAATTAATTCGATTTATTGCAAAATAGAGGATTGCGTAACACTTTCTGTTCAGAGTTTTCACGCAATTTCAATTTTGCCTTAATATTTTAATAACTTGTATCTTTCATCCTTGTTTTCATTTATCTTGTAAAAATAATTTACAAAGCCAGAATTCTTAGAGTATAATAGGTATCGAAACAATTTTTCAGAAAAATAAAAAAGGGAGGTCTACTTA |
| lmo2638 | 30 | GTTTTGCTCCCACCTTTTGTTTTATTGATTAACTTTGTTTGTGATTTCGTGAACACAAACCGATAGTCTGCGAAAGCGATATCACTTTTACAATACAAATGATACCATCAGCGCCCGGTTTTGTCTACTGTAATTCTAAAGACATAATTCATCTAATTTTCAAAATAATGACTATTGATATACAAAAGATGGCTTAAACCAACCTTTTCTAGCAAAATAGCAAACAAAAATGTCTAACATCCTATGTTAAACAAGGACTTTTAGCCTGCTTAATTATGTAACATTTGCACAAAGTCCTATTTTTCAAATTTAGATTAGAATTGTTCTTTACTATCTAGACATTAGTAGGAGGTTTCTGTTAAACTATGATAGGTTATAATAATACTGTGGGGAATTTTGGACTCATTTTATTTTTGTGAATAAACTTACAAGGAGTTGAGGAAGCAT |
| lmo2610 | 31 | GGAGTGAGCTGTGCTGAATCACTGTCAAAATCAATGCGTGCAAATTAGATTAGTTTGTAGTATAATGGATAATTGGCAGTGGAATTAAAGGCATCTAAGCAAATCATGATGAACGAGAGAACCGTTTGAAGATCATCCGAATGACGTATATGTTCTACCTTGATCTGTTCAGCCGCAATGCTTTTGCGAGTGACAGTTCTTCTGGTTTTTACGGATTTCACTTTTACAGGTTGTCTGAAAAGCTGGCGAAATGTGTTGCCTTTTAATGAAGTGCATCCATTTTTGCATGTTTTACGTTTTTTTCATGTAAGAGAAATAGTTACACTGAAAGGATTAGGCCTGCATCAGGAACAAATCCAAGAATACAAGAAGGAGGTAGCAAACAT |
| lmo0248 | 32 | AAAAATCTTGCAACTATACATTTAAAATGGTATCATATATAAGTACGTTTTTGACGTATGTTTTAACGTGGGAGGGGAAATATCAGCCCCAGTCAACCACATCACGGACTTAAGGAGGTATGTCTC |
| lmo2615 | 33 | TAATGTTAGTTCCTAAACGTGTAAAATACCGTCGTGAATTCCGCGGAAACATGCGTGGACGCGCGAAAGGCGGAACTGAAGTTGCATTTGGTGAATATGGTCTTCAAGCAGTTGAAGCTTCTTGGATTACAAACCGTCAAATCGAAGCAGCTCGTATCGCAATGACTCGTTACATGAAACGTGGCGGTAAAGTTTGGATTAAAATTTTCCCTCATAAATCTTACACTTCTAAACCAATCGGGGTTCGGATGGGTAAAGGTAAAGGTGCTCCGGAAGGTTGGGTAAGCCCAGTCAAACGTGGCAAAATTATGTTTGAAATCGCAGGTGTTCCTGAAGATGTAGCGCGTGAAGCATTACGTCTAGCAGCACACAAACTGCCGGTCAAAACTAAGATCGTTAAACGTGAAGAAATTGGTGGTGAAGCAA |
| lmo0582 | 34 | TATATAACATCCTCCATACCTTCTATTATAGAATACCATAAACTCATCTGGCAATTCATTTCGAGTCACGAAGAACGGAAAAACTGCCGGTTTTTATATTACAAATGTATTAAGTTTTTCTATTAACAAAAAACAATAGGTTTCCCATAGCGAAAGTTGTTGATTAACGTTCACATCCCACTTACACTATAAAGGTTTACCCAGCAGTACATCTCAAGCCCTAAGAATACACGTTCGCTTTTCAACTGTTACAGAATTATTACAAATAGTTGGTATAGTCCTCTTTAGCCTTTGGAGTTATTATCTCATCATTTGTTTTTTAGGTGAAAACTGGGTAAACTTAGTATTAATCAATATAAAATTAATTCTCAAATACTTAATTACGTACTGGGATTTTCTGAAAAAAGAGAGGAGTTTTATGAAT |
| lmo0202 | 35 | CGACGATAAAGGGACAGCAGGACTAGAATAAAGCTATAAAGCAAGCATATAATATTGCGTTTCATCTTTAGAAGCGAATTTCGCCAATATTATAATTATCAAAAGAGAGGGGTGGCAAACGGTATTTGGCATTATTAGGTTAAAAAATGTAGAAGGAGAGTGAAACCCATG |
| P_help_ | 36 | GATCTCGAGATCTGCAGGATCCCATTATGCTTTGGCAGTTTATTCTTGACATGTAGTGAGGGGGCTGGTATAATCACATACGGCCGATAAAGCAAGCATATAATATTGCGTTTCATCTTTAGAAGCGAATTTCGCCAATATTATAATTATCAAAAGAGAGGGGTGGCAAACGGTATTTGGCATTATTAGGTTAAAAAATGTAGAAGGAGAGTGAAACCCATG |


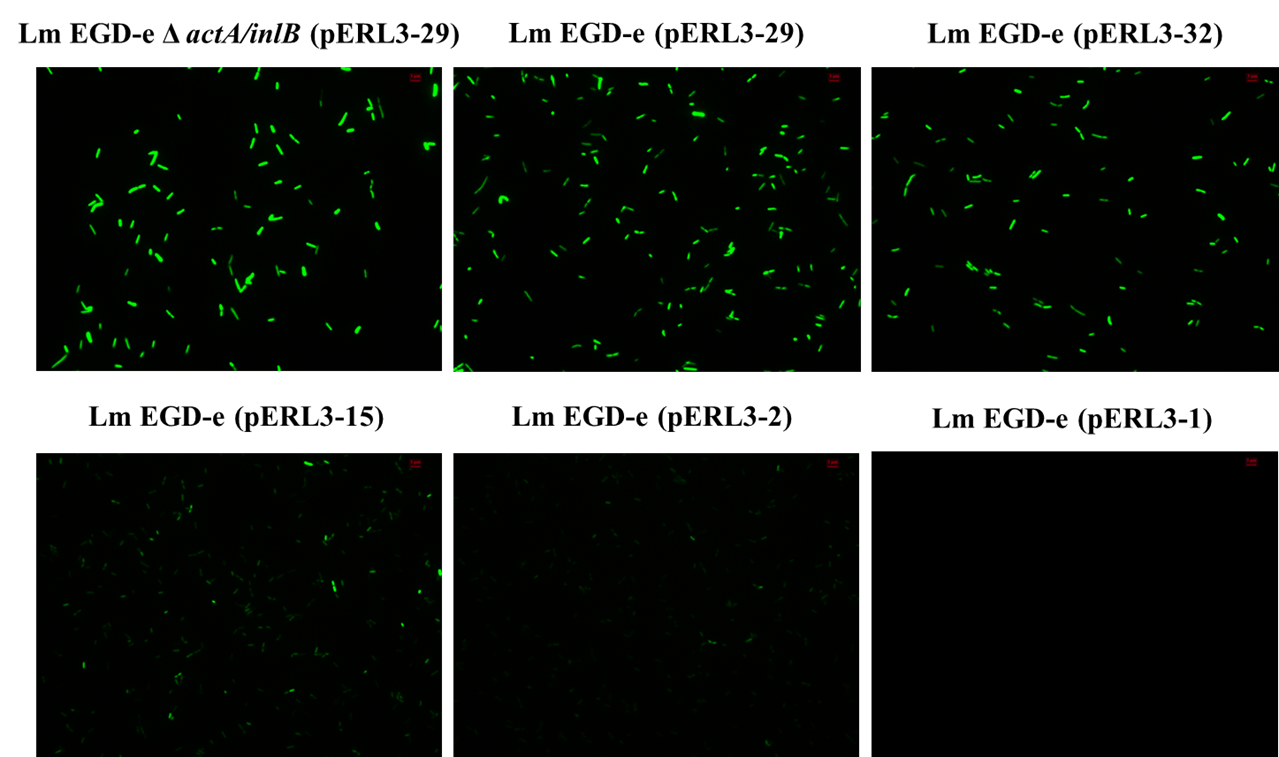


Fig. S1. Visualization of EGFP expression in Lm at 37 °C under fluorescence microscope.
